# Scheduling models and primary health care quality: a multilevel and cross-sectional study

**DOI:** 10.11606/S1518-8787.2019053000940

**Published:** 2019-04-26

**Authors:** Tiago Barra Vidal, Suelen Alves Rocha, Erno Harzheim, Lisiane Hauser, Charles Dalcanale Tesser

**Affiliations:** I Universidade Federal de Santa Catarina. Programa de Pós-Graduação em Saúde Coletiva. Florianópolis, SC, Brasil; II Universidade Estadual Paulista. Programa de Pós-Graduação em Saúde Coletiva. São Paulo, SP, Brasil; III Universidade Federal do Rio Grande do Sul. Faculdade de Medicina. Programa de Pós-Graduação em Epidemiologia. Porto Alegre, RS, Brasil; IV Universidade Federal do Rio Grande do Sul. Programa de Pós-Graduação em Epidemiologia. Porto Alegre, RS, Brasil

**Keywords:** Appointments and Schedules, Primary Health Care, organization & administration, Health Care Quality, Access, and Evaluation, Multilevel Analysis, Agendamento de Consultas, Atenção Primária à Saúde, organização & administração, Qualidade, Acesso e Avaliação da Assistência à Saúde, Multilevel Analysis

## Abstract

**OBJECTIVE:**

To evaluate whether the scheduling model influences the perception of the user about the quality of primary health care centers.

**METHODS:**

This is a cross-sectional and population-based study that measured the quality of centers by the Primary Care Assessment Tool (PCATool-Brazil), applied to adult users (n = 409) from 11 health centers in Florianópolis, state of Santa Catarina. Multilevel analysis was used to verify the relationship between the score of general quality of the primary health care and the scheduling model. The independent variables (age, skin color, scheduling model, panel size by primary health team, poverty ratio as income proxy, number of health teams, presence of economically interest areas, number of medical appointments in one year per primary health team, number of people treated in one year per health team), with p < 0.20 were selected for the multilevel model, which was adjusted with aggregates of information from users and health centers.

**RESULTS:**

The health center that used advanced access had a general score of 7.04, while those using a weekly carve-out had a score of 6.26; the carve-out every 15 days, score of 5.87; and the traditional carve-out, score of 6.29.

**CONCLUSIONS:**

The scheduling model of advanced access had a positive effect on the quality of primary health care, in the perception of users.

## RESUMO

**OBJETIVO:**

Avaliar se o modelo de agendamento influencia a percepção do usuário sobre a qualidade dos serviços da atenção primária à saúde.

**MÉTODOS:**

Estudo transversal de base populacional que mediu a qualidade dos serviços pelo instrumento *Primary Care Assessment Tool* (PCATool-Brasil), aplicado aos usuários adultos (n = 409) de 11 centros de saúde em Florianópolis, SC. A análise multinível foi utilizada para verificar a relação entre o escore de qualidade geral da atenção primária à saúde e o modelo de agendamento. As variáveis independentes (idade, cor da pele, modelo de agendamento, população por equipe de saúde da família, proporção de pobreza como *proxy* de renda, número de equipes de saúde da família, presença de áreas de interesse social, número de consultas médicas em um ano por equipe de saúde da família, número de pessoas atendidas em um ano por equipe de saúde) com p < 0,20 foram selecionadas para o modelo multinível, que foi ajustado com agregados de informações dos usuários e dos centros de saúde.

**RESULTADOS:**

O centro de saúde que utilizava acesso avançado apresentou o escore geral 7,04, enquanto os que utilizavam *carve-out* semanal tiveram escore 6,26, *carve-out* quinzenal escore 5,87 e tradicional escore 6,29.

**CONCLUSÕES:**

O modelo de agendamento acesso avançado teve um efeito positivo na qualidade da atenção primária à saúde, na percepção dos usuários.

## INTRODUCTION

The access, primary attribute of the quality in health care – access to structures and care processes in a timely manner, receiving effective care^1^ – directly influences the quality of primary health care (PHC).

In the last ten years, there has been a significant increase in population coverage by the PHC, mainly by the Family Health Strategy model (*Estratégia Saúde da Família*). However, the decentralization in the implementation and management of the PHC allowed a great variation in the quality of the health centers around the country. In addition, the greater availability of PHC centers did not result in an expressive improvement that ensured universal and equitable health coverage^2^.

Organizational changes are needed to address the issue of long delay in scheduling an appointment at PHC, which represents the inability of health centers to provide timely access and leads to avoidable negative experiences for users. The uncertainty about being seen or not causes distress, especially among those who believe that their health condition is progressively worsening. In addition, it contributes to the increase in the inappropriate use of emergency services^3^.

Improvements to the scheduling model can increase access to PHC^4^. An effective scheduling process improves the work environment, quality of care, patient safety, satisfaction of health teams, timely access, and user experience^5^. In the Methods section of this article, there is a characterization of the three scheduling models used (advanced, carve-out and traditional access). Other authors have already described the main current scheduling models^5–7^.

The implementation of advanced access is the most common and cost-effective intervention to reduce the delay in scheduling a PHC appointment^8^. In addition, this model is associated with the improvement of the satisfaction of the health team^10^ ^,^ ^11^, the longitudinality of patient care and safety^11^, as well as the reduction of absenteeism^9^ ^,^ ^10^, of inappropriate appointments in emergency services^10^ ^,^ ^11^, of medical time dispensed in the risk classification to prioritize appointments^10^ and of the negative work *backlog*, which consists of schedules resulting from the work not completed by the team in a same day, either because of the low supply or the organization of the work process itself^8^ ^,^ ^9^.

In advanced access, the user can obtain a appointment within two business days (but can be scheduled for later if the user prefers)^12^; there is no distinction between urgent (unscheduled) and routine (scheduled) appointments^6^, and the longitudinality of care is prioritized^12^. This system presupposes five principles: balance between supply and demand, reduction of negative backlog, improvement of interprofessional practices, contingency plan and revision of the scheduling system^6^.

In Brazil, since 2014, several advanced access deployment experiments have been conducted locally. This intervention was not included in any national orientation, and several initiatives were undertaken by the PHC teams in an attempt of meeting local demands. In addition, it has not yet been evaluated by any study.

This article aims to investigate the relationship between the quality of PHC perceived by the user and the appointment scheduling model adopted in the health center. To this end, the study evaluated whether PHC teams that promote advanced access had a higher PHC score (in essential and derivative attributes) than those using another appointment scheduling model.

## METHODS

Population-based cross-sectional study held in the health district in Northern Florianópolis in 2012. It used the Primary Care Assessment Tool (PCATool-Brasil) for adults as the instrument for measuring the PHC quality.

Adults (≥ 18 years) living in the territory covered by the 11 health centers (HC) of the region (n = 598) were included. Interviewees who did not have as reference service one of the health centers under analysis (n = 189) were excluded.

The interviewers were community health agents (n = 83) trained for this purpose, under the supervision of nurses (n = 24) of the Family Health Strategy teams, which is the PHC model of organization recommended by the municipal health policy. To minimize gauging biases, the community health agents did not make the interview in their own working area, being allocated to the area of another health center. A pilot of the collection was held for training and clarification of doubts about the application of the instrument. Field collection lasted five months (from May to September 2012).

The data that subsidized the identification of the appointment scheduling model were obtained through contact with each PHC, which was classified into one of the following models:

1. Advanced access: approximately 65%–90% of daily medical appointments are reserved for unscheduled care^6^ ^,^ ^7^. Most prescheduled appointments result from positive work backlogs, representing patients who do not want the appointment on that day and those who are scheduled by the physician after evaluation of clinical and social criteria. The maximum delay in booking a medical appointment is two business days.
2. Weekly carve-out: 50% of daily medical appointments are reserved for the care of unscheduled care, whereas 50% are for the care of scheduled appointments. The maximum delay in booking a medical appointment is five business days.
3. Carve-out every 15 days: 50% of daily medical appointments are reserved for the unscheduled care, whereas 50% are for the care of scheduled appointments. The maximum delay to booking an appointment is ten business days.
4. Traditional: all daily medical appointments are pre-booked (supersaturated schedule). There is no reserve of vacancies for unscheduled care, which are embedded between the appointments, generating double-booking. In this model the average delay in booking a medical appointment is quite variable, sometimes taking longer than thirty days.

### Instrument

The PCATool-Brasil^13^ evaluates the quality of PHC by the presence and extension of its essential (first contact access, comprehensiveness, longitudinality and care coordination) and derivative (family orientation and community orientation) attributes, through questions about health centers which can be answered by users, professionals or public administrators. This instrument was created based on the Donabedian quality assessment model, considering the measurement of aspects of health center structure, processes and outcomes^13^.

Stein^14^ describes the importance of using validated instruments to evaluate the quality of care in the PHC and improve its performance, besides stating that the PCATool is used by public administrators and researchers due to its excellent measurement properties. In Brazil it is currently the most widely used instrument to evaluate the effectiveness of family health teams^15^. The validation and use of this instrument in different countries, such as Canada, United States, Spain, China, Argentina and Brazil, shows its suitability in different sanitary and cultural contexts^16^.

All key attributes of PHC are measured by the PCATool-Brasil from the perspective of individual experience, including components related to the structure and the care process. The responses are of Likert type, with the scale ranging from one (“absolutely not”) to four (“absolutely yes”) and the additional option nine (“I do not know”/“I do not recall”). It has 87 items divided into 10 components related to PHC attributes:

1. degree of affiliation with the health center;
2. first contact access (sub-dimension utilization);
3. first contact access (sub-dimension accessibility);
4. longitudinality;
5. coordination (sub-dimension integration of care);
6. coordination (sub-dimension information system);
7. comprehensiveness (sub-dimension services available);
8. comprehensiveness (sub-dimension services provided);
9. family orientation;
10. community orientation.

According to the validation instrument PCATool-Brasil for adults, scores are standardized for a scale from zero to 10, with values equal to or greater than 6.6 considered as high, which corresponds to responses for options three or four on the instrument’s original scale. The standardization for the scale from zero to 10 is conducted as follows:

$$S\tan dardized\;score\;=\frac{(\mathrm{Escore}\;-\;1)\;\times \;10}{\left(4\;-\;1\right)}$$

The PHC quality score was calculated according to the PCATool-Brasil guidebook, adult version*,* of the Brazilian Ministry of Health. The general score (GS) of PHC, calculated as the mean of all attributes plus the degree of affiliation (mean value of essential and derivative attributes and degree of affiliation) was calculated^13^.

## Secondary Data

Secondary data from InfoSaúde, a computerized system used by the Municipal Health Secretariat (SMS) of Florianópolis were used. They covered the period from January 1, 2011 to December 31, 2011. All 11 health centers in the Northern health district during the period analyzed were included in the study.

Data on the presence or absence of economically deprived areas were obtained from the Department of Health Geoprocessing of the SMS in Florianópolis. Areas classified as economically deprived comply with the following criteria: low family income, housing and infrastructure network precariousness, environmental precariousness and risk and precarious areas in land tenure, and urban equipment and services^17^.

Data on the poverty ratio, defined as *per capita* income up to a minimum wage, were based on the nominal income of the population according to the SMS of Florianópolis. Other secondary data extracted from InfoSaúde were: panel size (population enlisted by health center), number of PHC teams per health center, number of medical appointments per year, and number of people attended per doctor per year. It should be noted that the study considered the period when some teams stood without a doctor during the period analyzed.

## Sampling and Statistical Analysis

To calculate the sample size, the OpenEpi^®^ software was used. A total of 459 questionnaires were required. Considering percentage of loss plus the estimated sample size of 30%, 598 questionnaires were applied. The parameters used for this calculation were: 95% confidence level, 5% absolute precision, and 1.2 design effect for the cluster effect adjustment to estimate the proportion of users that would assign high PHC scores (≥ 6.6) for 50% of the evaluated health centers.

The household sampling process was by clusters stratified by health center and distributed proportionally by their panel size. Households were selected by systematic sampling by street and house.

Descriptive analysis was then performed with absolute frequency, percentage, mean and standard error. The multilevel analysis methodology was used to observe the relationship between the dependent variable (general PHC score) and the study factor (scheduling model adopted).

First, adjustments were made to the model using the dependent variable, the factor under study and the other independent variables in an individual way: age, race, poverty ratio (used in the study as a proxy income variable), panel size per PHC team, number of PHC teams, economically deprived area, number of medical appointments in the year and number of people assisted in the year. The independent variables that presented p-value less than 0.20 in this first model were chosen to compose the final multilevel model. Although the variable age was not statistically significant, it was maintained in the multivariate model for the purpose of adjusting estimates because of its conceptual relevance.

The multilevel model was adjusted according to the aggregates of information: individual (users of health centers) and health centers. The results were presented by the coefficient B, its respective confidence intervals (95%CI) and p-values. The significance level of 5%, bilateral, was used for all statistical analyses. The suitability of the model was verified using residue analysis and the presence of collinearity between the variables. The Box shows the two-level model: the first shows the characteristics of users and the second shows the characteristics of health centers.

**Box:** Conceptual model used for multilevel analysis.

Level 1
Characteristics of users
– Age
– Sex
– Skin color
– Poverty ratio (income proxy variable)

Level 2

Characteristics of Health Centers
– Scheduling model
– Panel size by health team
– Presence of economically deprived areas
– Number of medical appointments in one year per health team
– Number of people served in one year per health team

General PHC score (degree of orientation for PHC)

PHC: primary health care

The interviewees participated voluntarily in the research and signed an informed consent form, from which they received a copy, according to Resolution 466/2012 of the National Health Council. The research project was approved by the Monitoring Committee of the Municipal Health Research Projects and by the Research Ethics Committee Involving Human Beings of the Universidade Federal de Santa Catarina, which issued a favorable opinion under number 1,635,663.

## RESULTS

A total of 598 users were interviewed. The proportion of users who attributed a high PHC score to the health centers evaluated was 46.45% (n = 190), in line with the proportion used to calculate the sample size. Approximately 70% of the people (n = 409) reported using health centers as a reference service for primary health care. This was the final sample of the subsequent analyses.

Table 1 presents the description of health centers in the northern health district, with secondary data collected and general PHC score according to PCATool-Brasil. Only 36.36% of the health centers evaluated had a high general PHC score, the highest being the health center that used advanced access.

**Table 1:** Description of health centers in the Northern sanitary district according to PCATool-Brasil in 2012. Florianópolis, state of Santa Catarina, 2017.

Health centers	Scheduling model	Number of health teams	Panel size by health team (inhabitants)	Presence of economically deprived areas	Number of medical appointments in one year per FH team	Number of people served in one year per health team	General PHC score* measured by PCATool-Brasil based on evaluation of users
1	Weekly	3	5,249	Yes	2,908	1,525	6.48
2	Advanced	5	3,784	No	4,433	1,533	7.05
3	Every 15 days	3	4,579	No	2,663	1,270	5.39
4	Weekly	2	5,651	Yes	3,760	1,264	6.01
5	Weekly	2	3,581	No	3,125	1,250	7.23
6	Traditional	1	6,910	No	3,480	1,155	5.68
7	Traditional	1	4,114	No	2,989	1,606	6.10
8	Traditional	1	1,630	No	3,231	593	6.71
9	Traditional	1	2,828	No	3,486	1,696	6.86
10	Every 15 days	2	2,746	Yes	2,554	987	6.19
11	Traditional	1	4,160	Yes	2,970	1,362	6.09

PCATool: Primary Care Assessment Tool; PHC: primary health care

* Score ranging from 0 to 10, representing the mean of the score among all individuals interviewed who reported having the health center evaluated as a referral service.

Table 2 presents the distribution of the characteristics of the users according to the scheduling model. The general mean age of the interviewees was 47 years, with a standard deviation (SD) of 0.86. Regarding skin color, 91.9% declared themselves white.

**Table 2:** Distribution of the characteristics of users according to the scheduling model. Florianópolis, state of Santa Catarina, 2017.

Characteristic	General	Scheduling model									

		Traditional (n = 63)			Weekly carve-out (n = 160)		Carve-out every 15 days (n = 90)			Advanced access (n = 96)	
				
	Mean (SD)	Mean (SD)	Min.-Max.		Mean (SD)	Min.-Max.	Mean (SD)	Min.-Max.		Mean (SD)	Min.-Max.
Age (years)	47.0 (0.86)	48.4 (2.4)	(16.0–85.0)		46.2 (1.3)	(16.0–80.0)	46.5 (1.6)	(18.0–89.0)		48.5 (2.2)	(20.0–78.0)

	n (%)										

Skin color											
White	376 (91.9)	59 (93.7)	-	143 (89.3)		-	85 (94.0)	-	89 (92.2)		-
Non-white	33 (8.1)	4 (6.3)	-	17 (10.7)		-	5 (6.0)	-	7 (7.8)		-

SD: standard deviation; Min.-Max.: minimum–maximum

Table 3 shows the mean general PHC score in health centers according to the scheduling model. It was verified that the health center that adopted the advanced access presented high mean general PHC score, whereas the others presented low score.

**Table 3:** Mean general score* of primary health care measured by PCATool-Brasil of health centers according to the scheduling model. Florianópolis, state of Santa Catarina, 2017.

Scheduling model	Mean	Standard error	95%CI
Advanced access	7.04	0.49	6.09–8.00
Weekly carve-out	6.26	0.27	5.67–6.74
Carve-out every 15 days	5.87	0.35	5.18–6.57
Traditional scheduling	6.29	0.27	5.67–6.74

PCATool: Primary Care Assessment Tool

* Score ranging from 0 to 10.

Table 4 shows the characteristics associated to the general PHC score, as well as the results of the adjusted models using the multilevel methodology. The variable poverty ratio was dichotomized by its median, whose result was 11%. Considering the models with adjustment of the independent variables to the outcome, it can be observed that the scheduling model and the panel size were shown to be associated with the mean general PHC score.

**Table 4:** Characteristics associated to the general primary health care score in the adult users’ perception users of health centers. Florianópolis, state of Santa Catarina, 2017.

Characteristic	Univariate model*			Multivariate model*		
	
	Beta	95%CI	p	Beta	95%CI	p
Users						
Age (increase by age group every 10 years)	0.04	-0.05–0.13	0.40	0.04	-0.05–0.13	0.41
Skin color						
White	0.19	-0.33–0.70	0.48			
Non-white	0.00					
Health centers						
Scheduling model						
Weekly carve-out	-0.49	-1.60–0.61	0.38	-1.41	-2.53–-0.30	0.01
Carve-out every 15 days	-1.16	-2.33–0.01	0.03	-2.36	-3.61–-1.10	0.00
Traditional scheduling	-0.89	-1.98–0.19	0.11	-2.64	-4.24–-1.05	0.00
Advanced access	0.00			0.00		
Panel size per health team (every 1,000 individuals)	-0.26	-0.51–0.00	0.05	-0.11	-0.20–-0.02	0.01
Proportion of poverty (income) (median = 0.11)						
Up to 0.11	0.45	-0.22–1.13	0.19			
More than 0.11	0.00					
Number of health teams (increase of 1 FH team)	0.14	-0.15–0.43	0.35			
Economically deprived areas						
Presence	0.07	-0.85–0.98	0.88			
Absence	0.00					
Number of appointments in one year per health team (every 100 appointments)	0.04	-0.02–0.10	0.20			
Number of people served in one year per FH team (every 100 people)	0.00	-0.13–0.14	0.97			

* Adjusted through multilevel methodology (individual-level and contextual-level variables).

In the multilevel multivariate model, there was a statistical significance of the difference in the general mean PHC score of the health center with advanced access in relation to the others. It also allowed the inference of an inversely proportional relationship between the delay in scheduling appointments and the mean general PHC score, using the advanced access as a parameter to compare the different scheduling models. PHC teams using the traditional model obtained a mean general PHC score worse than that of the carve-out model every 15 days, which in turn had a general mean PHC score worse than that of the weekly carve-out model (β = -2.64; β = -2.36; β = -1.41, respectively). Therefore, all these values of beta (β) were negative, that is, they represent lower general mean PHC scores when compared to the advanced access, keeping constant the age of the users and the panel size.

In addition, the results showed that the greater panel size of a PHC team, the lower their general PHC score, keeping constant the age and the scheduling model. The results show that the increase of 1,000 people reduces, on average, 0.11 in the general mean PHC score.

## DISCUSSION

The main result of this article was to identify the association between the scheduling model and the general mean PHC score in Florianópolis. Most of the studies on advanced access point to the increasing productivity and the reducing absenteeism in this scheduling model in comparison to the others^9^, not addressing the quality of health services.

The multilevel analysis shows that the health center adopting the advanced access obtained a higher general mean PHC score than the health centers with other scheduling models. PHC users expect timely access and high-quality health services. The existing literature shows that advanced access is associated with improved quality of medical appointment^18^, care provided^19^ and clinical results of diabetic users^20^, with a decrease in the delay of medical appointments^9,21–23^ and absenteeism^9^ ^,^ ^21^.

It was also verified that a longer delay in scheduling appointments is inversely related to the general PHC score. Negative health outcomes are associated with longer delay periods in scheduling PHC appointment. In addition, O’Hare and Corlett^19^, as well as Lukas et al.^24^, reported an increase in user satisfaction after reducing delay in scheduling appointments, reinforcing the importance of timely access to PHC centers.

The panel size by PHC team was inversely related to the general PHC score. Large panels in PHC centers are associated with lower quality of disease prevention and health promotion activities, poorer management of chronic diseases, lower technical quality of care provided, and negative interference in effective access and longitudinality of care. However, there are no studies on the effect of panel size on health equity^25^. The new National Policy of Basic Care^26^ (PNAB – *Política Nacional de Atenção Básica*), of 2017, recommends a panel size of 2,000 to 3,500 users per family doctor. According to Murray and Tantau^6^, it should be approximately 2,500 people. In England, Kiran et al.^27^ suggest reducing this number per professional, from 2,500–3,550 to 1,800 people. In the USA, Peterson et al.^28^ identified that about 50% of the total number of family doctors questioned (n = 11,231) and who devoted 81 to 100% of their time in direct clinical care had a panel of 1,501 to 3,000 users, most of whom (21.3%) reported having between 1,501 to 2,000 users. Masseria et al.^29^ described the organization of PHC in 14 European countries and found that the average panel size per general practitioner is 2,000 users, except in Poland (4,161 users). Therefore, it is observed the indication of panels smaller than those recommended by the current PNAB in socioeconomic contexts less adverse, iniquitous and consequently pathogenic than the Brazilian ones^30^.

A key requirement for the deployment of advanced access is the balance between supply and demand in PHC centers. To achieve this balance, two organizational measures are recommended: reduction of the delay in scheduling an appointment and adjustment of the panel size. Considering that in the international literature these two measures are associated with timely access, it seems that the association found between the advanced access and the quality perceived by the users is due to the fact that this scheduling model increases the timely access in the PHC centers evaluated.

Among the limitations of this study is the fact that it does not measure the number of appointments and of people attended by registered nurses, only medical appointments. Another limitation was not to evaluate among the different scheduling models of medical appointments: absenteeism, unmet demand, working time of the health team and time of professional training and qualification, as well as the workday of the professionals. These variables would be useful in the analysis and interpretation of the results found.

## FINAL CONSIDERATIONS

This study detected a positive effect of the advanced access to PHC quality. It is expected and understandable that the shift to scheduling models that are more agile and sensitive to the needs of users is positively related to the quality of care provided in PHC centers.

Organizational changes aimed at improving the performance and quality of PHC should include measures that are easily understood by the population and the health team and that have been successfully tested in similar scenarios. For this, it is fundamental to verify the relation between interventions and the presence and extension of the PHC attributes as a measure of PHC quality. Other researches in distinct Brazilian contexts and scenarios are necessary to corroborate the findings reported here.
